# Soil Eukaryotic Microorganism Succession as Affected by Continuous Cropping of Peanut - Pathogenic and Beneficial Fungi were Selected

**DOI:** 10.1371/journal.pone.0040659

**Published:** 2012-07-10

**Authors:** Mingna Chen, Xiao Li, Qingli Yang, Xiaoyuan Chi, Lijuan Pan, Na Chen, Zhen Yang, Tong Wang, Mian Wang, Shanlin Yu

**Affiliations:** Shandong Peanut Research Institute, Qingdao, China; Virginia Tech, United States of America

## Abstract

Peanut is an important oil crop worldwide and shows considerable adaptability but growth and yield are negatively affected by continuous cropping. Soil micro-organisms are efficient bio-indicators of soil quality and plant health and are critical to the sustainability of soil-based ecosystem function and to successful plant growth. In this study, 18S rRNA gene clone library analyses were employed to study the succession progress of soil eukaryotic micro-organisms under continuous peanut cultivation. Eight libraries were constructed for peanut over three continuous cropping cycles and its representative growth stages. Cluster analyses indicated that soil micro-eukaryotic assemblages obtained from the same peanut cropping cycle were similar, regardless of growth period. Six eukaryotic groups were found and fungi predominated in all libraries. The fungal populations showed significant dynamic change and overall diversity increased over time under continuous peanut cropping. The abundance and/or diversity of clones affiliated with *Eurotiales, Hypocreales*, *Glomerales*, *Orbiliales, Mucorales* and *Tremellales* showed an increasing trend with continuous cropping but clones affiliated with *Agaricales*, *Cantharellales*, *Pezizales* and *Pyxidiophorales* decreased in abundance and/or diversity over time. The current data, along with data from previous studies, demonstrated that the soil microbial community was affected by continuous cropping, in particular, the pathogenic and beneficial fungi that were positively selected over time, which is commonplace in agro-ecosystems. The trend towards an increase in fungal pathogens and simplification of the beneficial fungal community could be important factors contributing to the decline in peanut growth and yield over many years of continuous cropping.

## Introduction

Changes in micro-organism populations in soils are critical to the sustainability of soil-based ecosystem function because of their involvement in such key processes as mineral nutrition cycling, organic matter turnover,soil structure formation and toxin removal or accumulation [1], [2]. The productivity and sustainability of agricultural soil have a substantial affect on plant growth and final crop quality and both are significantly affected by the diversity and abundance of soil micro-organisms [3], [4].

Microbial community structures in soils are affected by the treatment or management of the soil. Amendment with fertilizer [5], application of pesticides [6], different tillage regimes [7] and biological amendments, such as the introduction of genetically modified micro-organisms [8], [9], have all been shown to affect soil microbial community structures. Soil type, crop selection and cropping sequence are key determining factors on soil micro-organisms [10]–[12]. Due to their quick response to environmental changes, micro-organisms are seen as efficient bio-indicators of soil condition and land management success [13], [14].

Soil micro-organisms are also related to soil borne plant diseases and plant disease suppression. Fungi (true fungi and oomycetes) and nematodes are major causes of soil borne diseases [15] and considerable research attention has been paid to potential bio-control agents against plant fungal pathogens in recent years [16]–[18]. Many fungal populations and bacterial groups have been identified as antagonists of soil borne plant pathogens and play important roles in promoting plant growth [19]–[21]. Interestingly, it has been shown that several bacterial groups suppress soil borne plant pathogens through synergistic relationships with arbuscular mycorrhiza fungi [22], [23]. The mechanisms by which these micro-organisms suppress plant disease can be divided into several categories: nutrient competition, amensalism, microbial antagonism, parasitism and systemic induced resistance [10]. In addition, it has been suggested that fungal diversity also has a beneficial effect on plant pathogen suppression and plant productivity [24]. Because of the interaction between soil micro-organisms and soil borne plant disease, the assessment and management of soil microbial community structure are central to the successful suppression of plant pathogens [25].

Due to the relationships between micro-organisms, soil quality and plant health, active management of soil microbial communities such as the manipulation of microbial populations as linked to agricultural production [26] could be a promising approach to improving soil conditions, developing natural suppression of soil borne diseases and improving crop productivity. However, relatively little is known about the specific populations, characteristics, interactions, and relationships between plants and soil micro-organisms, especially the fungal plant pathogens and their antagonists.

Peanut, one of the four most important oil crops, can adapt to a wide range of climatic conditions, and grows in many different environments between latitudes 40°N and 40°S [27]. However, its productivity drops significantly when subjected to continuous cropping. In addition, the yield of other main world crops, such as wheat, rice, corn and soybean, also decline under continuous cropping [28]–[30]. The negative impact of continuous cropping on soil productivity has been shown to have a significant effect on soil microbial biomass and community structure [3], [31], [32]. However, this decline is not yet well understood. A comprehensive knowledge of specific inhabitants, characteristics, soil microbial succession and how soil micro-organisms interact with continuous cropping practices is still lacking. This is particularly true for eukaryotic soil micro-organisms. Sudini *et al*. [11] studied bacterial communities over two peanut cropping cycles, but there have been no relevant reports on eukaryotic micro-organisms to date.

This investigation is a first attempt to study the succession of eukaryotic soil micro-organisms under continuous peanut cultivation. This was achieved by using cultivation-independent 18S rRNA gene clone library analyses. The goals of this study were to: (i) determine the phylogenetic affiliations of the most common and predominant populations of soil microbes at various peanut growth stages under a continuous cropping regime; (ii) identify the diversity and succession patterns of eukaryotic micro-organisms, with particular emphasis on the fungi.

## Materials and Methods

### Management Description and Soil Sampling

The study was conducted between 2008 and 2010 at Shandong Peanut Research Institute in Qingdao, China (36.10°N, 120.41°E). Peanuts were planted in May each year and harvested in October. Pot culture experiments were undertaken with forty seedlings each year. The pot diameter was 0.45 meter. The soil consisted of 28.5% clay, 41% loam, and 30.5% sand, with no known history of peanut cropping. In order to reduce interference from outside factors on the soil microbial community and truly reflect the relationship between soil eukaryotic microbial succession and continuous cropping with peanut: two rain-proof plastic buildings with two opposite opening sides were built, the plants were irrigated with sterile water and no fertilizer was applied during the growing seasons and fallow periods.

Samples were collected at four different peanut growth stages in 2008: at the seedling stage, full-flowering stage, pod bearing stage and pod-maturing stage. In 2009 and 2010 samples were only collected at the seedling stage and pod-maturing stage. The soils were sampled using a soil probe (1.5 cm diameter) from individual plant at 5–10 cm soil depths, and at distances of 3–5 cm, 8–10 cm and 13–15 cm from the main root, respectively. These distances were chosen because roots at this depth and these distances were relatively abundant and the soil microbial community around the peanut root system could be well characterized. In addition, external interference due to the sampling of the under-surface layer soil could be reduced. At each stage, five randomly selected replicates with six cores in each (two cores for each distance) were pooled together to form a homogenous sample. The samples were stored at −80°C and were used for eukaryotic micro-organism analysis.

### Genomic DNA Extraction and 18S rRNA Gene Library Construction

DNA was extracted from the pooled material for each sample with Ultra-Clean soil DNA kits (MoBio, USA). The quality and quantity of the genomic DNA extracted were determined by electrophoresis on 0.8% (wt/vol) agarose gels. Primers, nu-SSU-0817 and nu-SSU-1536 [33], were used for 18S rRNA gene amplification. The reaction was performed for 25 cycles using the ‘Easy-A High-Fidelity’ PCR master mix from Stratagene (USA). Products from six PCRs were pooled, purified, using a Gel Extraction kit (Omega, USA), and ligated into pMD19-T simple vectors (Takara, Japan). The hybrid vectors were cloned into *Escherichia coli* TOP10. Clone libraries were constructed by random selection of approximately 110 white colonies from a single plate for each library. All clone libraries were named on the basis of the plant growth stage in the crop cycle and time of soil sampling. 08S, 08F, 08B and 08M were libraries constructed respectively for the seedling stage, full-flowering stage, pod-bearing stage and pod-maturing stage in 2008; 09S and 09M were libraries constructed respectively for the seedling stage and pod-maturing stage in 2009 and 10S and 10M were libraries constructed respectively for the seedling stage and pod-maturing stage in 2010.

### 18S rRNA Gene Sequencing and Data Analysis

Cloned 18S rRNA gene fragments were re-amplified by using primers RV-M (5′-GAGCGGATAACAATTTCACACAGG-3′) and M13-D (5′-AGGGTTTTCCCAGTCACGACG-3′) that flanked the insertion site of the cloning vector. All positive clones were sequenced to improve data accuracy and integrity.

Operational taxonomic units (OTUs) in each library were defined by using the DOTUR program at the 3% distance level [34]. Chimeric sequences were identified as described by Berney *et al.* [35]. The library coverage was calculated as follows:[1−(n1/N)] ×100, where n1 is the number of unique OTUs (frequency, 1) detected in the library and N is the total number of clones in the same library [36]. The value obtained approximately corresponded to the species’ coverage level in a given sample. Sampling efficiency and the sequence phylotype diversity of the libraries were analyzed using the rarefaction curve method and the aRarefactWin program [36].

Similarities between the soil eukaryotic assemblages at different stages of peanut growth during different crop cycles were established by performing cluster analyses on the constructed libraries using the software package SPSS 11.5 (SPSS, United States). The frequency (*F*) for the OTUs was calculated as follows: *F* = (*m*/*N*) × 100, where *m* is the clone number of an OTU in a library and *N* is the total number of clones in the same library. Log-transformed frequency data were used to minimize the influence of preferential PCR amplification [37].

The nearest phylogenetic neighbor sequence for each OTU sequence was determined in the GenBank database using the BLASTN program [38]. Sequences were aligned using ClustalX v.1.81 [39]. Phylogenetic trees were constructed using the DNADIST and NEIGHBOR programs in the PHYLIP package (version 3.65c) [40] and bootstrap support was evaluated using 100 replicates.

### Nucleotide Sequence Accession Numbers

The 18S rRNA gene sequences determined have been deposited in the GenBank database under accession numbers JQ320555 - JQ321347.

## Results

### Statistical and Comparative Analyses for the Eight Libraries

Eight 18S rRNA gene libraries were constructed over three continuous cropping cycles and representative growth stages of peanut. A total of 841 clones were screened, and 802 of them contained a proper insert. A total of 125 OTUs were identified, nine of which (10 clones) were probably chimeras, based on the chimera-check results. The library coverage ranged from 77.8 to 89.7% (Table 1). Rarefaction analysis confirmed the OTU phylotype diversity of the soil eukaryotic micro-organisms (Figure S1). The analysis results showed that the 116 valid OTUs accounted for most of the microbial eukaryote diversity present in the soil samples studied.

**Table 1:** Analysis of the clone libraries constructed over three continuous cropping cycles and representative growing stages of peanut.

Library	No.of clones	No. of total OTUs	No. of unique OTUs	% Coverage	No. of OTUs sharedby libraries fromsame cropping cycle	% shared OTUs
08S	99	29	12	87.9	10	34.5
08F	98	29	14	85.7		34.5
08B	100	25	13	87.0		38.5
08M	97	26	10	89.7		38.5
09S	99	35	22	77.8	18	51.4
09M	96	33	19	80.2		54.5
10S	103	38	20	80.1	22	57.9
10M	101	34	22	78.2		64.7

The coverage values were calculated as follows: [1−(n1/N)]×100, where n1 is the number of unique OTUs (frequency, 1) detected in the library and N is the total number of clones in the same library.

**Figure 1:**
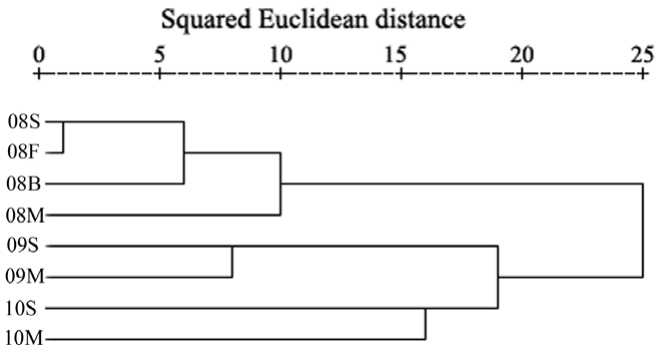
Clustering analysis of the 18S rRNA gene clone libraries. Dendrogram based on a hierarchical clustering analysis of the 18S rRNA gene clone libraries, constructed using the squared Euclidean distance similarity and Ward linkage procedures.

Phylotype diversity increased during continuous cropping with peanut. The OTU number ranged from 25 to 29 in the four libraries for the first cropping cycle; was 35 and 33, respectively, in the second cycle libraries and was 38 and 34, respectively, in the third cycle libraries (Table 1). In addition, clone libraries obtained from the same cycle but from different growth stages typically shared 10 to 22 common OTUs, accounting for 34.5 to 64.7% of the OTUs in each library (Table 1). The dynamics of the microbial eukaryotic community were further confirmed by cluster analyses. The hierarchical (Figure 1) and k-means (data not shown) clustering results were consistent and showed that libraries obtained from different growing stages, but from the same cropping cycle, were grouped together. The four clone libraries (S1, F1, B1, M1) obtained from the first cycle were grouped into a class, the two libraries (S2, M2) obtained from the second cycle were grouped into a second class, and the two libraries (S3, M3) from the third cycle were grouped into a third class. These results demonstrated that the eukaryotic soil microbial populations changed when peanut was grown continually, regardless of growth stage.

### 18S rRNA Gene Sequence Analyses

All the 116 valid OTUs were used for sequence analysis, most (77.6%) of which showed high levels of similarity (≥97% identity) with known sequences in the GenBank database and 7.0% were moderately related (≤94% identity) to known sequences. Phylogenetic analysis showed that 95 of the OTUs were affiliated with fungi, which predominated in all the libraries, accounting for 86.9 to 94.1% of obtained clones in each library (Table S1). The remaining 21 OTUs were related to five groups: *Alveolata*, *Metazoa*, *Rhizaria*, *Stramenopiles*, and *Viridiplantae* (Table S1). The phylotype diversity of fungi increased under continuous cropping. OTU numbers ranged from 19 to 25 for the four measurements in the first cropping cycle libraries, 29 and 28 in the second cycle libraries and increasing to 30 and 29 in the third cycle libraries. Apart from the population dynamics of the predominant fungi, the *Stramenopiles* and *Rhizaria* also showed succession changes with continuous cropping. OTUs affiliated with *Stramenopiles* only occurred in the four libraries of the first cropping cycle. Corresponding sequences had 92 to 97% similarity to known GenBank sequences and were moderately related to genera of *Nerada* and *Rhizidiomyces*. However, only OTUs related to *Rhizaria* occurred in the four libraries covering the 2009 and 2010 continuous cropping cycles. Sequences for the second and third cropping cycles had 92 to 98% similarity to known sequences and were moderately affiliated with genera of *Cercomonas*, *Lecythium*, *Paracercomonas* and *Rhogostoma*, all of whom belonged to *Cercozoa*.

**Figure 2:**
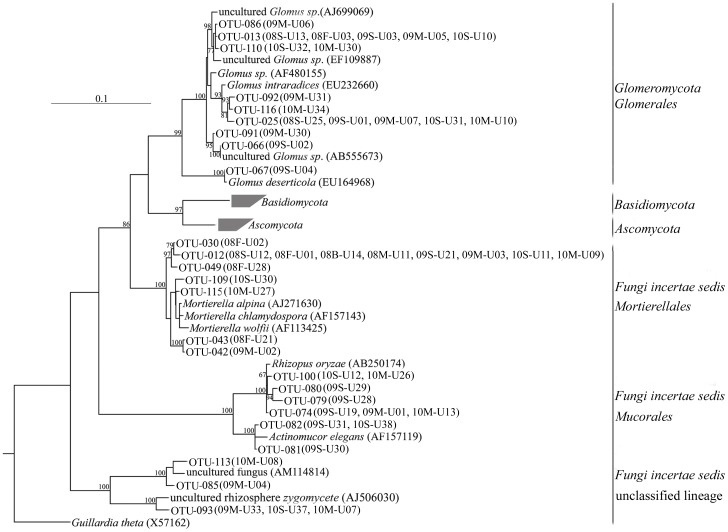
Phylogenetic tree of the *Fungi* sequences. Phylogenetic tree of the *Fungi* sequences recovered from the eight 18S rRNA gene clone libraries, constructed using the neighbor-joining method with the Kimura two-parameter model for nucleotide change. The *Ascomycota* and *Basidiomycota* which were just denoted the phylogenetic positions in this tree, presented in other separate phylogenetic trees. The libraries OTUs occurred were labeled. Scale bar, 0.1 substitutions per nucleotide position. Bootstrap values (100 replicates) above 60% are indicated at the nodes. The tree was rooted using the sequence related to *Guillardia theta* (X57162).

Four fungal groups were identified from the clone libraries, including the following phyla or divisions: *Ascomycota*, *Basidiomycota*, *Fungi incertae sedis* and *Glomeromycota* (Figure 2). The phylum *Ascomycota* dominated all the libraries accounting for 47.2% of the total 793 clones (excluding the 10 chimeric clones) and showed the greatest diversity, accounting for 38.8% of the 116 valid OTUs. *Basidiomycota* were also relatively abundant, accounting for 30.5% of the obtained valid clones and were also highly diverse, accounting for 21.6% of OTUs analyzed. The *Fungi incertae sedis* group and *Glomeromycota* phylum were not so abundant and diverse, accounting for 8.8% and 4.8%, respectively, of the 793 clones analyzed and each accounted for 13.8 and 7.8%, respectively, of the valid OTUs characterized. However, they both showed an increase in abundance and diversity as the number of cropping cycles increased.

### 1) Sequences of the *Ascomycota*

**Figure 3:**
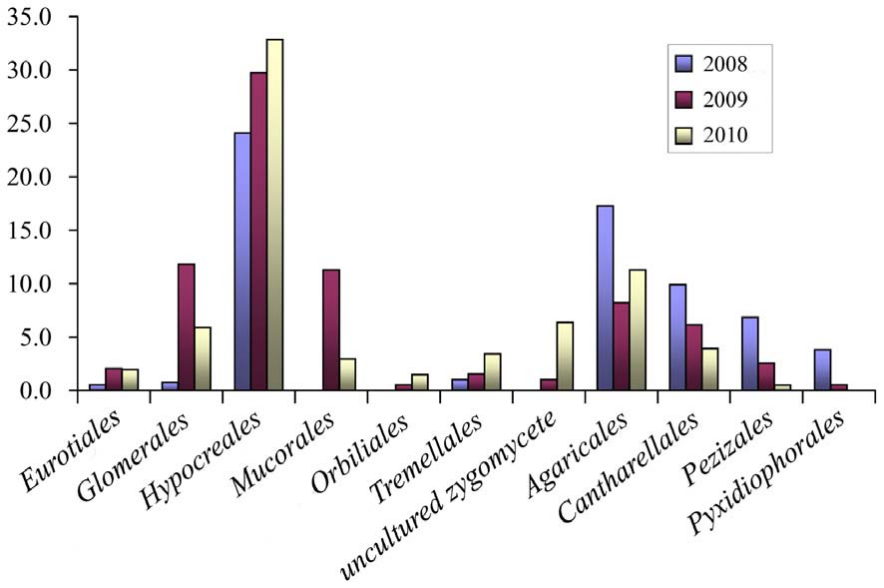
The clone abundance analyses of the fungal orders or phylotypes. The clone abundance analyses of the fungal orders or phylotypes which showed succession changes in populations with continuous cropping cycles. (The clone abundance was calculated as: (*m*1/*M* × 100, where *m*1 is the number of related clones detected in the libraries for the same cropping cycle and *M* is the total number of clones in the same libraries.).

Forty five OTUs (47.2% of the total clones) were affiliated to *Ascomycota*, which predominated in all the libraries and showed the greatest diversity. Seven classes were affiliated with the *Ascomycota* phylum and these were: *Dothideomycetes*, *Eurotiomycetes*, *Laboulbeniomycetes*, *Leotiomycetes*, *Orbiliomycetes*, *Pezizomycetes* and *Sordariomycetes*. There were 13 correlative orders or subdivisions presented in the libraries (Figure S2). The *Hypocreales* sequences were most abundant and diverse, accounting for 58.8% of the *Ascomycota* clones and for 31.1% of the corresponding OTUs. The *Eurotiales* and *Pezizales* sequences were relatively diverse, accounting for 6.7% and 11.3% of the OTUs affiliated with *Ascomycota*, respectively. Comparison of the three cropping cycle libraries indicated that the OTU diversity and/or abundance of the three orders, *Eurotiales*, *Hypocreales* and *Orbiliales*, increased over the period of continuous cropping, but the order *Pezizales* and *Pyxidiophorales* decreased significantly in diversity and/or abundance (Figure 3 and Figure S2).

The *Hypocreales* sequences predominated in all the analyzed libraries, accounting for 24.1, 29.7, and 32.8%, respectively, of the the first, second and third cycle library clones (Figure 2). The sequences also showed great diversity, accounting for 12.1% of the 116 OTUs analyzed. These sequences had a high similarity level to known sequences (95–99%) and were related to genera of *Acremonium*, *Bionectria*, *Fusarium*, *Geosmithia*, *Paecilomyces*, *Trichoderma, Cordyceps* and an uncultured *Hypocreales* genus. The most common and abundant *Hypocreales* OTUs belonged to *Fusarium*, which was represented in all eight libraries and accounted for 88.2% of the 220 *Hypocreales* clones detected in the libraries. Clone abundance accounted for 22.1, 26.7 and 27.0% of the first, second and third cycle libraries, respectively. Five OTUs were related to *Bionectria*, and accounted for 0.5, 2.1 and 4.4%, respectively, of the clones obtained in the three cycle libraries. OTU-001 affiliated with *Fusarium solani* with a 98% similarity and was common in all clone libraries. The other OTUs related to *Hypocreales* only occurred in one or two of the libraries. OTUs related to *Paecilomyces* and *Acremonium* were only found in the first cycle libraries; OTUs related to the uncultured *Hypocreales* genus, were found in the second and third cycle libraries, but not in the first cycle libraries, and OTUs related to *Geosmithia* and *Trichoderma* were only found in the library S2 and M3, respectively.

Three and two OTUs, respectively, were related to the order of *Eurotiales* and *Orbiliales*. The analysis indicated that the two order phylotype diversity both increased with continuous peanut cropping, despite of low abundance. Only one OUT related to *Eurotiales* occurred in the first cycle libraries, and two OTUs occurred in the second and third cycle libraries, respectively. OUT related to *Orbiliales* only were found in the second and third cycle libraries, but not found in the first cycle libraries. The clone abundance of the two orders also showed the same tendency. The *Eurotiales* clones accounted for 0.5, 2.1, and 2.1% of the first, second and third cycle library clones, respectively. These sequences had 98 to 99% similarity to known sequences, including genera of *Aspergillus* and *Penicillium*. *Penicillium* clones (OTU-077) occurred in the libraries of the second and third continuous cropping cycles, but not in the first cycle libraries. The *Orbiliales* clones accounted for 0, 0.5 and 1.5% of the first, second and third cycle libraries. The sequences had 97 to 98% similarity to known sequences, including genera of *Orbilia* and an uncultured *Orbiliaceae* clone.

Six OTUs (33 clones) were related to the order *Pezizales.* Both the diversity and abundance of these clones decreased over time with continuous peanut cropping. Four OTUs were found in the first cycle libraries, two OTUs were found in the second cycle libraries and only one OTU was found in the third cycle libraries, which only occurred in the peanut initial growth library S3 and not in the pod-maturing stage library M3. In addition, the *Pezizales* clones accounted for 6.8, 2.6, and 0.5% of clones in the first, second and third cycle libraries, respectively. These sequences had 95–99% similarity to known Genbank sequences and were related to four genera: *Chalazion*, *Marcelleina*, *Peziza* and *Sarcosphaera*. The *Chalazion* clones were present in all the first cycle libraries, but were not found in any second and third cycle libraries. Clones related to *Peziza* occurred in five libraries, which were the four first cycle libraries and the initial growth library of the second cycle (library S2). The *Sarcosphaera* clones only occurred in the S1 library. In contrast, clones related to *Marcelleina* only occurred in the second and third cycle libraries and only in libraries for the initial growth stages S2 and S3.

Only one OTU (16 clones) belonged to the order *Pyxidiophorales* and this was 98% related to *Pyxidiophora arvernensis*. The results showed that this OTU mainly appeared in the four first cycle libraries and only one clone was found in the M2 library.

### 2) Sequences from the *Basidiomycota*

The *Basidiomycota* OTUs were relatively abundant and diverse in the libraries, with 25 OTUs accounting for 30.5% of the 793 valid clones that were affiliated. These OTUs were affiliated with *Agaricomycetes* and *Tremellomycetes.* The *Agaricomycetes* OTUs made up the majority of the members of the *Basidiomycota* detected in the libraries, accounting for 88.0% of the related OTUs and 94.2% of the related clones. Four orders or phylotypes were present: *Agaricales*, *Cantharellales*, *Geastrales* and *Sebacinales* (Figure S3). OTUs related to the class *Tremellomycetes* detected in the libraries were only affiliated with the order *Tremellales*. When the three cycle libraries were compared, it was found that the phylotypes of *Agaricales*, *Cantharellales* and *Tremellales* showed the great dynamic population changes (Figure3 and Figure S3).

The *Agaricales* sequences were the most dominant *Basidiomycota* phylotype in the libraries, accounting for 44.0% of the 25 OTUs and 44.2% of the 242 related clones. *Agaricales* clone abundance significantly decreased in the second cycle libraries compared to the first cycle libraries but there were no significant differences between the second and third cycle libraries. The *Agaricales* clones accounted for 17.3% of the clones in the first cycle libraries but only 8.2, and 11.3% of the clones in the second and third cycle libraries, respectively. Clone diversity also showed a similar tendency. There were 10 OTUs identified in the first cycle libraries but only three OTUs identified in the second and third cycle libraries, respectively. These sequences had high similarity to known sequences (96–99%) and were affiliated with the genera *Camarophyllopsis*, *Coprinus, Hemimycena, Panaeolus* and *Psathyrella*. OTU-016 (50 clones), affiliated with the genus *Psathyrella,* was the most common and abundant OTU, appearing in all eight libraries. OTUs from the remaining genera showed considerable dynamic population changes and occurred in between one and five libraries. OUT-028 related to *Camarophyllopsis* only occurred in the library S1; OTUs related to *Panaeolus* occurred in the first and third cycle libraries; OTUs related to *Coprinus* only occurred in library F1, B1 and S2 and OTUs related to *Hemimycena* occurred in most of the first cycle libraries, only occurred in the second library M2, but not occurred in any third library.

Only five OTUs were affiliated with the order *Cantharellales,* but were relatively abundant, accounting for 24.4% of the *Basidiomycota* clones. The clone abundance decreased significantly with continuous cropping, accounting for 9.9, 6.2 and 3.9% of the clones detected in the first, second and third cycle libraries, respectively. These sequences were associated with genus of *Ceratobasidium, Rhizoctonia* and *Thanatephorus* with 96 to 99% similarity. OTU-017 affiliated with *Thanatephorus* was common in all eight libraries. The other four OTUs only appeared in one or two libraries.

In contrast to the decreasing population trend shown by the *Agaricales* and *Cantharellales*, the *Tremellales* showed an increasing population trend with continuous cropping. Despite only three OTUs (14 clones) being related to *Tremellales,* clone abundance still showed a significant increase accounting for 1.0, 1.5, and 3.4% respectively, of the first, second and third cycle library clones. These OTUs were all affiliated with genus *Cryptococcus* with 99% similarity. OTU-26 was relatively common, appearing in six libraries except B1 and M3, but the other two OTUs only occurred in the third cycle libraries.

### 3) Sequences of the *Fungi Incertae Sedis*

Sixteen OTUs (70 clones), accounting for 13.8% of the 116 valid OTUs, were affiliated with the *Fungi incertae sedis* where there were two classes or subdivisions, *Mucoromycotina* and an unclassified lineage, present. The *Mucoromycotina* sequences were the predominant members of the *Fungi incertae sedis* detected in the libraries, accounting for 78.6% of the characterized clones. Two orders related to *Mucoromycotina* were identified, including *Mortierellales* and *Mucorales*. Three OTU (15 clones) were affiliated with an early diverging fungal lineage and were unclassified. Comparison analysis indicated that phylotypes related to *Fungi incertae sedis* changed significantly in the three libraries over the three continuous cropping cycles (Figure 1 and Figure 3).

The *Mortierellales* and *Mucorales* OTUs were relatively abundant, accounting for 43.8 and 37.5% of the *Fungi incertae sedis* OTUs and for 38.6 and 40.0%, respectively, of the 70 related clones. The *Mortierellales* OTUs were common in all eight libraries, and were all affiliated with the genus *Mortierella* with 98–99% similarity. Clone abundance showed no significant change over the three cycle libraries and only OUT-012 was common, the other six OTUs occurred in only one library. The *Mucorales* OTUs occurred in all four libraries covering the second and third cropping cycles but were not found in any first cycle library. The clone abundance rose and fell over the three year cropping period, accounting for 0, 11.3 and then 2.9% of the clones detected in the first, second and third cycle libraries, respectively. These sequences were affiliated with genera of *Actinomucor* and *Rhizopus* with 98–99% similarity. Three OTUs were affiliated with an early diverging fungal lineage and these were related to uncultured *zygomycete*. OUT-093 began to appear in the second cycle library at the pod-maturing stage (library M2). Clone abundance increased successively accounting for 1.0, 3.9 and 7.9% of the clones identified in libraries M2, S3 and M3, respectively. The other two OTUs only occurred in the library M2 and M3, respectively.

### 4) Glomeromycota Sequences

Nine OTUs (38 clones) were affiliated with the *Glomeromycota,* with 97–99% similarity to known GenBank sequences, and all sequences were related to the genus *Glomus* belonging to the order *Glomerales*. Analysis results indicated that clone diversity and abundance both increased significantly with continuous peanut cropping (Figure 1 and Figure 3). However, they slightly decreased in the third cycle libraries compared to the second cycle libraries. The OTU number was 2, 1, 4, 5, 3 and 3 in libraries S1, F1, S2, M2, S3 and M3, respectively, but they did not occur in the later growth stage libraries, B1 and M1. The clones accounted for 2.0, 1.0, 13.1, 10.4, 6.8 and 5.0%, respectively, of the clones detected in the corresponding libraries. This result indicated an increasing trend in related clone abundance with continuous peanut cropping but a decrease in peanut growth with each cropping cycle.

## Discussion

In this study, 18S rRNA gene clone library analyses were undertaken to study the succession of soil eukaryotic micro-organisms in a soil under continuous peanut cultivation. In order to minimize the occurrence of artifacts and reduce the drawbacks of PCR-based rRNA analysis [41], homogenous samples were prepared and six PCR products were mixed for clone library construction. It is known that soil microbial communities are often difficult to fully characterize, mainly because of their considerable phenotypic and genotypic diversity as well as their heterogeneity [42]. In addition, most of them are generally unculturable also being a reason for that [43]. Based on the combination of rarefaction analysis and library coverage value calculations (ranging from 77.8 to 89.7%), these results show that most of the microbial eukaryotic phylotypes in the test soil, especially the common and dominant members, were accurately estimated using library analyses based on direct DNA extraction. Despite the disadvantages inherent in using this method [10], it does offer the possibility of assessing the total microbial diversity present by modulation.

Soil micro-organism communities are affected by a wide range of factors [44]. It has been reported that the treatment or management of soil, especially the soil type, the choice of plant species and cropping patterns, are the factors that most affect the microbial community structure in soil [10], [12]. In order to truly reflect the relationship between soil eukaryotic microbial succession and continuous cropping with peanut, three measures were employed to reduce interference from other factors, as described in the material and methods. Cluster analyses indicated that soil microbial eukaryotic assemblages obtained from the same cropping cycle were similar, regardless of growth stage. Phylotype diversity was also shown to increase with continuous cropping. These findings indicated that dynamic changes in soil microbial eukaryotic communities do occur with continuous peanut cropping. Soil microbial biomass and structure has also been found to be significantly influenced by continuous cropping with cucumber, rice and pea [3], [31], [32]. Different rotation sequences also had significant effects on soil microbial community structures [9], [32]. Therefore, it has been shown that microbial populations in soil are significantly selected by cropping patterns. However, the successional progress of specific microbial phylotypes and the interaction mechanisms between micro-organisms and plants need to be investigated further.

The data from this study showed that the fungal clones were the most common and dominant microbial eukaryotes in the soil during every growth stage in the three cropping cycles and the related phylotypes indicated significant dynamic changes in microbial populations with continuous cropping. It has also been reported that fungal biomass altered in continuously cropped rice fields [31] and arbuscular mycorrhizal fungi (AMF) declined in a continuous pea cropping regime relative to a pea–wheat rotation [3]. These results indicate that the soil fungal community was affected by continuous cropping and this may be a common phenomenon in agro-ecosystems.

Soil fungi have a high functional diversity. Some fungi improve soil quality and plant growth, such as AMF, by taking up nutrients and transferring them to the plants, thereby enhancing plant nutrition [45], [46]. Some have been identified as plant pathogens and impair plant growth while some others are antagonistic against pathogens [24], [47]. Consequently, it can be supposed that the changes in the fungal community with continuous cropping can disturb ecological function and balance, which may reduce growth over time.

Many fungal phylotypes identified in the libraries created by this study showed dynamic changes in populations with the continuous peanut cropping. The trends were complex and can be mainly divided into four types: trends showing a continuous increase or decrease; a trend where the population increases and then decreases or a trend where the population deceases and then increases. The complexity of microbial populations seen in this study raises many questions, such as what specific phylotypes were present? Why did populations change over time and what roles did the changing micro-organism population play during continuous cropping?

The abundance and/or diversity of clones affiliated with *Eurotiales, Hypocreales*, *Orbiliales* and *Tremellales* all increased with continuous cropping. The *Hypocreales* clones were common and predominant in all libraries and showed a high diversity. These diverse genera showed various trends during continuous cropping. *Fusarium* clones were abundant and ubiquitous and abundance increased in the second and third cropping cycles; Abundance of *Bionectria* clones also increased in the second and third cropping cycles. Clones related to an uncultured *Hypocreales* genus began to appear from the second cropping cycle but *Geosmithia* and *Trichoderma* clones did not appear until the third cycle. In contrast, clones related to genera *Acremonium* and *Paecilomyces* disappeared in the second cycle. Clones from the orders *Eurotiales* (genera *Aspergillus* and *Penicillium*) *Orbiliales* (genus *Orbilia*) and *Tremellales* (genus *Cryptococcus)* were not so diverse but their abundance increased with continuous cropping of peanut. Most of the related genera identified have been shown to be pathogenic to plants, including *Fusarium*, *Geosmithia*, *Acremonium*, *Aspergillus*, *Penicillium* and *Cryptococcus* [10], [17], [48]–[51]. In contrast, many species affiliated with *Trichoderma* and *Paecilomyces* have been reported to be suppressive of plant fungal pathogens [47], [52], [53]. The complex trends shown by these populations may imply complex interactions between them.

The relationships between pathogenic fungi and antagonists are complex. Competition, antagonism and hyperparasitism have been suggested as being possible interaction mechanisms [24], [54]. Beneficial micro-organisms have a wide range of mechanisms that inhibit the growth or activity of pathogenic micro-organisms. However, pathogens also have an array of mechanisms to counteract antagonists. An example of pathogen-antagonist interaction has been described for *Fusarium* and *Trichoderma asperellum*, *Trichoderma asperellum* isolates were suppressive to *Fusarium* wilt of tomato, but the mycotoxin, deoxynivalenol, produced by *Fusarium* species, acted as a negative signal repressing the expression of the nag1 chitinase gene in *Trichoderma atroviridae* [55], [56]. So the pathogen-antagonist interaction is mutual.

The dynamic population changes in *Mucorales* clones further suggests the complexity in interactions between pathogens and antagonists. Members of the order *Mucorales* have been identified as plant pathogens [57] and their related clones showed a relatively complex succession tendency. They were not found in any first cycle libraries; the abundance ratio rose to 11.3% in the second cycle libraries but then decreased to 2.9% in the third cycle libraries. This result, combined with the upper tendency analyses of pathogen related clones, all indicated that soil fungal pathogens increased under a continuous cropping regime, but the succession models were non-single.

The increase in fungal pathogens in the soil may result in more intense competition by some antagonistic fungi. *Glomerales* species are extensive root colonizers, and the genus *Glomus,* belonging to the AMF, has been shown to have the ability to protect host plants against pathogens [58], [59]. The abundance and diversity of *Glomus* clones increased significantly with continuous cropping in the first and second cycles but slightly decreased in the third cycle libraries. But clone abundance showed a decreasing trend with each subsequent cropping cycle. Analyses using clones related to pathogen antagonists predict a more complex dynamic succession of beneficial micro-organisms in soil under a continuous cropping regime.

Based on these analyses, it can be supposed that soil pathogens and antagonists first existed in a balanced equilibrium but under the stress of a continuous peanut cropping regime, the original pathogenic micro-organisms became more abundant, and as the soil micro-environment changed, new pathogens appeared and some original antagonists decreased or disappeared. At the same time, the original antagonistic inhabitants increased and very few new antagonist members appeared. This meant that few pathogen populations decreased or disappeared. However, in general, populations of fungal pathogens increased under continuous cropping.

Numerous diseases and symptoms are displayed by plants infected with soil borne fungal pathogens. However, these diseases are difficult to diagnose, because most of the symptoms occur below ground [60]. Consequently, the relationships between soil borne diseases and continuous cropping have been little reported but other recent studies have confirmed the results produced by this investigation. Root rot is the primary disease caused by soil borne pathogens [54]. It has been demonstrated that under a continuous pea, regime, root abundance was reduced two-fold compared to a pea–wheat rotation and *Fusarium* root rot was more severe [3]. This was consistent with the current analyses that *Fusarium* clone abundance increased with continuous peanut cropping. In addition, continuous cropping also increased the incidence of potato diseases, such as black scurf, stem canker and common scab [61], again implying an increase in plant pathogen populations, which is also consistent with the results from this study. These results suggest that the increasing trend in fungal pathogens related to *Eurotiales, Hypocreales*, *Mucorales* and *Tremellales* could be an important factor limiting the growth of peanut under a continuous cropping regime.

In contrast to the increase in the population of clones related to pathogens, the abundance and diversity of clones affiliated with *Agaricales* and *Pezizales* decreased under continuous peanut cropping. The *Agaricales* and *Pezizales* identified in the libraries were diverse. Most of the related genera, including *Chalazion, Peziza, Sarcosphaera*, *Camarophyllopsis*, *Coprinus* and *Hemimycena,* mainly occurred in the libraries from the first cropping cycle. The *Panaeolus* clone succession was unusual in that they existed in the first cycle, disappeared in the second cycle, and re-appeared again in the third cycle. The orders *Agaricales* and *Pezizales* have been reported to be beneficial fungi [62], [63]. Despite that, their roles in soil quality and plant growth are not well understood. Based on these fungi, it can be suggested that beneficial fungi decreased under continuous cropping and the recurrence of a few members like *Panaeolus* may be due to the recovery ability of the soil ecosystem. Simplification of the beneficial fungal community may also contribute to a decline in peanut growth under continuous cropping.

The results have highlighted the pathogenic and beneficial fungi that were significantly selected by continuous cropping and the overall trends that took place. There were dynamic population changes in the phylotypes present in the libraries. The *Cantharellales* and *Pyxidiophorales* clones identified were not so diverse, but were ubiquitous in every cropping cycle and continuously decreased over time. In addition, some clones were related to unclassified *Fungi incertae sedis* lineages. None of these clones existed until the late stage of the second cropping cycle and the clone abundance successively increased. Despite their unknown function, they must be connected with continuous cropping stress and should not be dismissed. They could be suppressed in more balanced environments but are able to thrive in environments influenced by intensive continuous cropping. Many phylotypes that decreased continuously, such as *Agaricales*, *Pezizales*, *Cantharellales* and *Pyxidiophorales,* could reflect genotype homogenization in the soil. The environmental and genetic uniformity of the agricultural ecosystem may promote the emergence of new pathogens [64], so these unclassified fungi that appeared after intensive mono-cropping, may be related to new pathogenic species.

The current study, along with previous studies, demonstrated that the soil microbial community was affected by continuous cropping; especially with regards to the pathogenic and beneficial fungi that were significantly selected for, and this process could be common in agro-ecosystems. The fungal populations were the most predominant and common members of the soil micro-eukaryotic populations and showed significant dynamic changes under continuous cropping, especially the pathogenic and beneficial fungi. The increase in the fungal pathogens and simplification of the beneficial fungal community could be important factors contributing to the decline in peanut growth under continuous cropping.

It is the first step toward fully understanding the microbe-soil-peanut interaction under continuous cropping stress. The goal of this kind of study is to manipulate or alter the soil microbial characteristics through various management practices that increase soil microbial activity, diversity, populations of plant-beneficial organisms, and pathogen antagonists, resulting in suppression of the negative factors that bring about the decline in plant growth under a continuous cropping regime. There are many related areas of research that need further investigation. For example, how do pathogenic and beneficial fungi interact with the soil, the plant and with one another? What roles do soil bacteria play in the microbial community? What management practices should be adopted so that the soil microbial characteristics can be beneficially altered or manipulated and which plant growth stages would most benefit from any changes?

## Supporting Information

Figure S1 **Rarefaction curves for the 18S rRNA gene libraries constructed from each of the soil samples.**
(TIF)

Figure S2 **Phylogenetic tree representing affiliations of the 18S rDNA sequences related to the** ***Ascomycota*** **phylum.**
(TIF)

Figure S3 **Phylogenetic tree representing affiliations of the 18S rDNA sequences related to the** ***Basidiomycota*** **phylum.**
(TIF)

Table S1 **The abundance and diversity analyses of clones affiliated with six eukaryotic groups determined in libraries.**
(DOC)
