# Supplementary figures and images for: Soil Eukaryotic Microorganism Succession as Affected by Continuous Cropping of Peanut - Pathogenic and Beneficial Fungi were Selected
Source: PLoS One. 2012 Jul 10;7(7):e40659. doi: 10.1371/journal.pone.0040659 (PMC3393692; doi:10.1371/journal.pone.0040659)

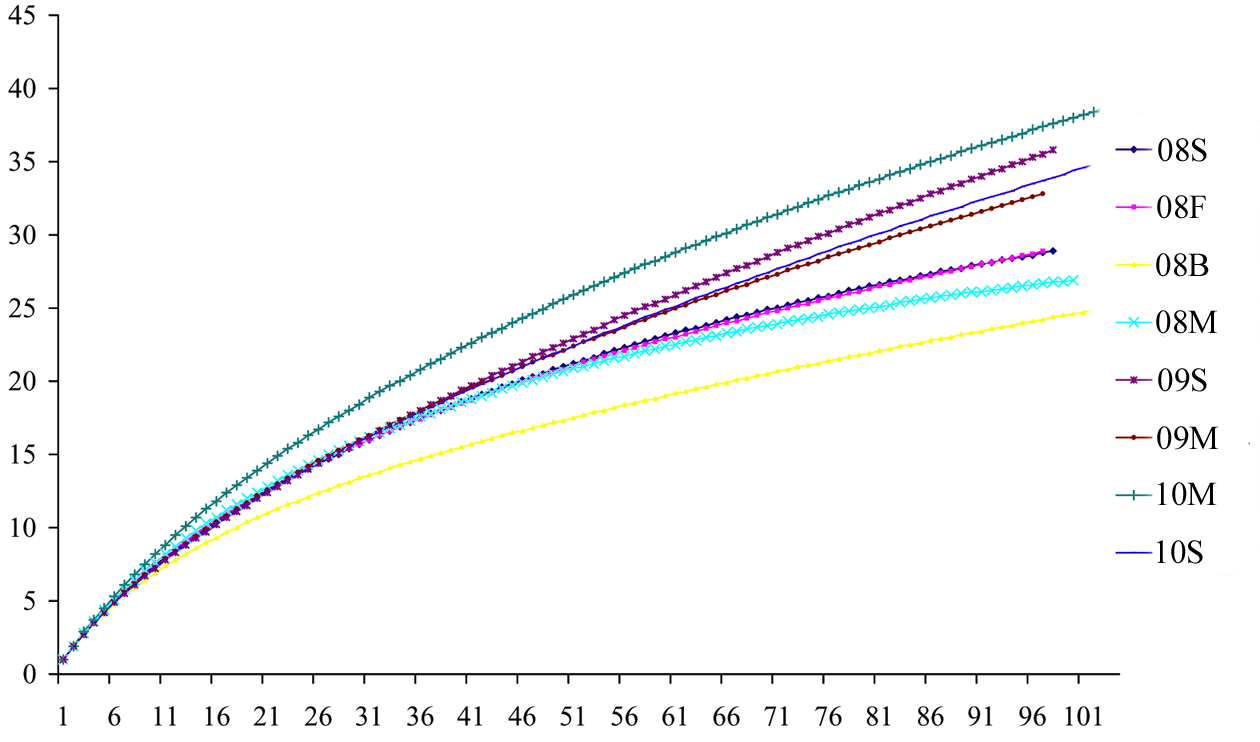

Supplement: Figure S1 — Rarefaction curves for the 18S rRNA gene libraries constructed from each of the soil samples. (TIF) [file pone.0040659.s001.tif]

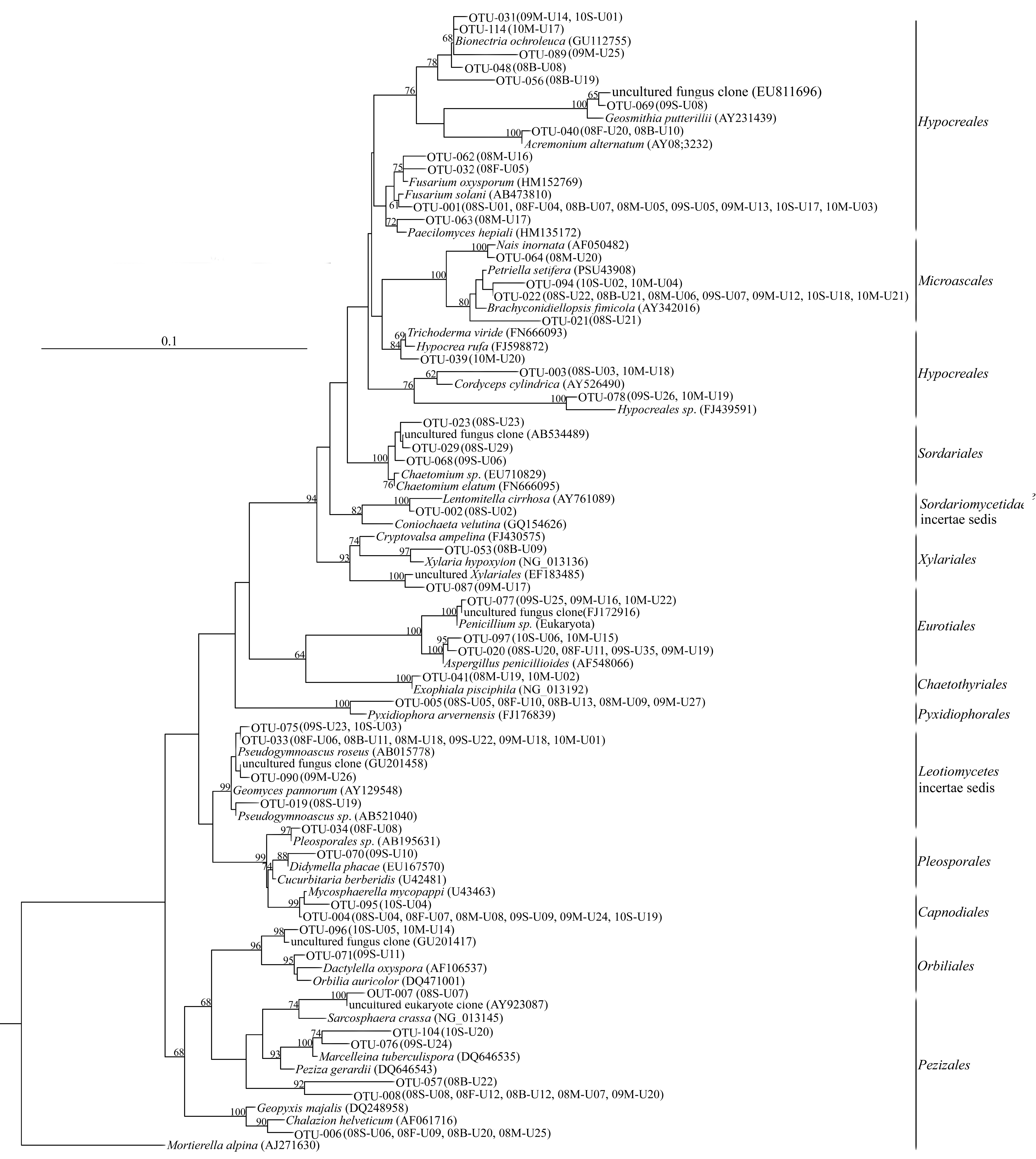

Supplement: Figure S2 — Phylogenetic tree representing affiliations of the 18S rDNA sequences related to the Ascomycota phylum. (TIF) [file pone.0040659.s002.tif]

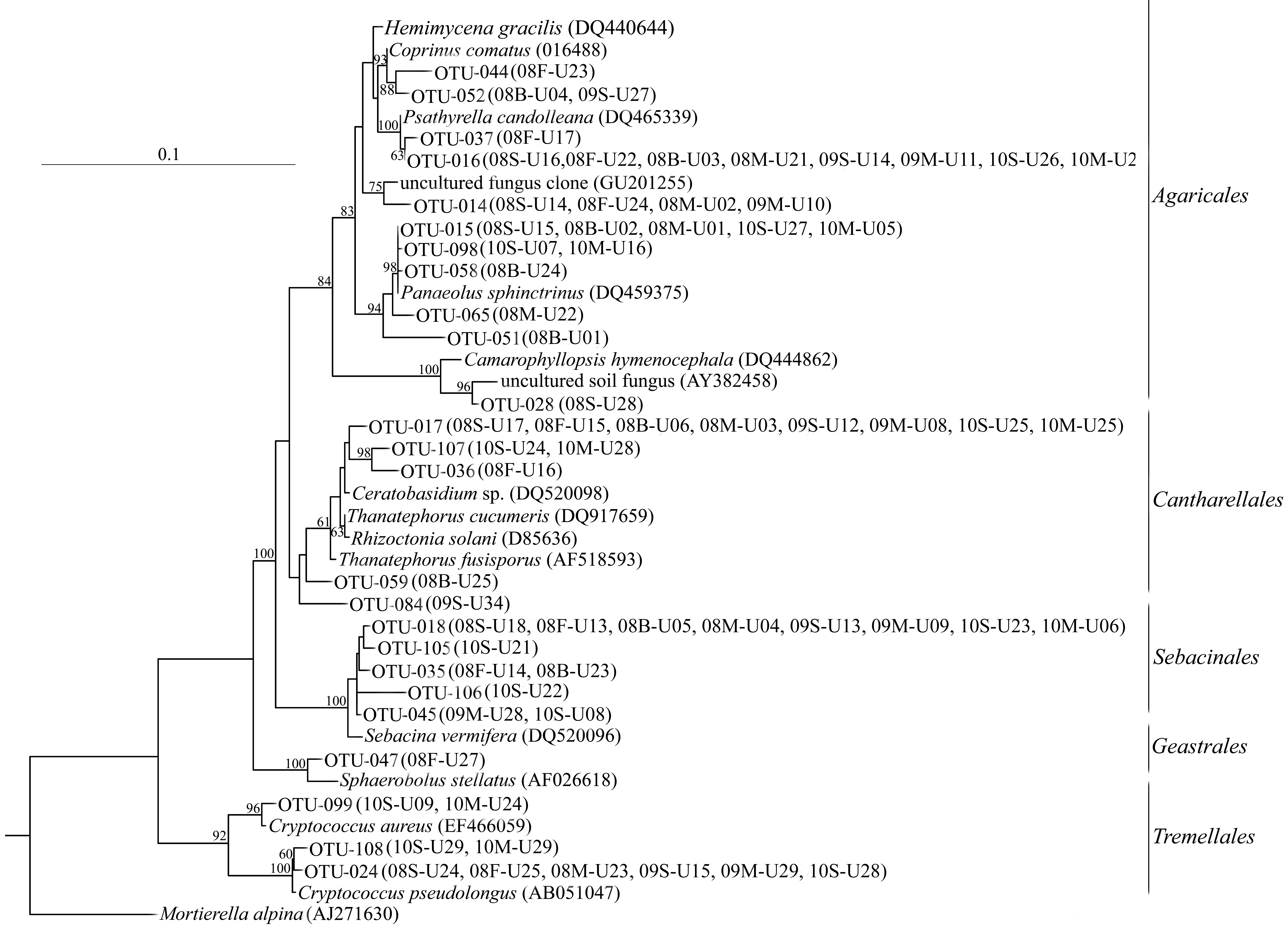

Supplement: Figure S3 — Phylogenetic tree representing affiliations of the 18S rDNA sequences related to the Basidiomycota phylum. (TIF) [file pone.0040659.s003.tif]
