# Supplementary material for: Soil Eukaryotic Microorganism Succession as Affected by Continuous Cropping of Peanut - Pathogenic and Beneficial Fungi were Selected
Source: PLoS One. 2012 Jul 10;7(7):e40659. doi: 10.1371/journal.pone.0040659 (PMC3393692; doi:10.1371/journal.pone.0040659)
Supplement: Table S1 — The abundance and diversity analyses of clones affiliated with six eukaryotic groups determined in libraries. (DOC) [file pone.0040659.s004.doc]

TABLE. S1. The abundance and diversity analyses of clones affiliated with six eukaryotic groups determined in libraries.

| Eukaryotic  groups | Percentage of clones | | | | | | | | Number of OTUs | | | | | | | |
| --- | --- | --- | --- | --- | --- | --- | --- | --- | --- | --- | --- | --- | --- | --- | --- | --- |
|  | 08S | 08F | 08B | 08M | 09S | 09M | 10S | 10M | 08S | 08F | 08B | 08M | 09S | 09M | 10S | 10M |
| *Fungi* | 86.9 | 92.9 | 91.0 | 89.7 | 91.9 | 93.8 | 90.3 | 94.1 | 24 | 25 | 21 | 19 | 29 | 28 | 30 | 29 |
| *Metazoa* | 6.1 | 2.0 | 3.0 | 4.1 | 1.0 | 1.0 | 1.0 | 1.0 | 2 | 1 | 2 | 3 | 1 | 1 | 1 | 1 |
| *Viridiplantae* | 5.1 | 4.1 | 5.0 | 3.1 | 1.0 | 2.1 | 3.9 | 1.0 | 1 | 2 | 1 | 2 | 1 | 1 | 2 | 1 |
| *Stramenopiles* | 2.0 | 1.0 | 1.0 | 3.1 | 0 | 0 | 0 | 0 | 2 | 1 | 1 | 2 | 0 | 0 | 0 | 0 |
| *Alveolata* | 0 | 0 | 0 | 0 | 0 | 0 | 1.9 | 0 | 0 | 0 | 0 | 0 | 0 | 0 | 2 | 0 |
| *Rhizaria* | 0 | 0 | 0 | 0 | 6.1 | 3.1 | 2.9 | 4.0 | 0 | 0 | 0 | 0 | 4 | 3 | 3 | 3 |
